# Subversion of the immune response of human pathogenic spirochetes

**DOI:** 10.1002/jcla.24414

**Published:** 2022-04-11

**Authors:** Jielite Huang, Jinlin Chen, Yafeng Xie, Zhuoran Liu

**Affiliations:** ^1^ Department of Clinical Laboratory The Second Affiliated Hospital, Hengyang Medical School, University of South China Hengyang China; ^2^ Institution of Pathogenic Biology Medical College Hunan Province Cooperative Innovation Center for Molecular Target New Drug Study University of South China Hengyang China

**Keywords:** immune evasion, immune response, persistent infection, spirochetes

## Abstract

Spirochetes are a large group of prokaryotes that originated from Gram‐negative bacteria and are capable of causing a variety of human and animal infections. However, the pathogenesis of spirochetes remains unclear, as different types of spirochetes play pathogenic roles through different pathogenic substances and mechanisms. To survive and spread in the host, spirochetes have evolved complicated strategies to evade host immune responses. In this review, we aimed to provide a comprehensive overview of immune evasion strategies in spirochetes infection. These strategies can be explained from the following points: (i) Antigenic variation: random, unidirectional, and segmental conversion of the gene to evade immune surveillance; (ii) Overcoming the attack of the complement system: recruitment of host complement regulators, cleavage of complement components and inhibition of complement activation to evade immune defenses; (iii) Interfering with immune cells to regulating the immune system; (iv) Persistent infection: invading and colonizing the host cell to escape immune damage.

## Graphical abstract

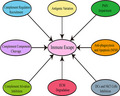

## 1 INTRODUCTION

Spirochetes are prokaryotic microorganisms that are slender, flexible, spiral‐shaped, and highly motile. They are widely distributed in nature and are commonly found in water, soil, and other putrefactive organic matter, as well as in the human mouth and animal body. Spirochetes are mainly divided into five genera of Spirochaetaceae based on differences in size, regularity, space structure, physiological characteristics, and host cells. There are namely *Cristispira*,^1^ *Spirochaeta*,^2^ *Treponema*,^3^ *Borrelia*,^4^ and *Leptospira*.^5^ Only *Treponema*, *Borrelia*, and *Leptospira* are known to cause diseases in humans (Table 1). Epidemiological studies have shown that the incidences of syphilis,^6^, ^7^ Lyme disease,^8^, ^9^ and leptospirosis^10^, ^11^ have increased rapidly worldwide and posed major threats to public health.

**TABLE 1:** The taxonomy and pathogenicity of spirochetes

Spirochaetaceae	Morphology	Length (μm)	Type species	Diseases	Pathogens	Medium
*Cristispira*	Ridged spiral	30–180	*Cristispira pectinis*	No	‐‐‐‐‐	Mud, sewage
*Spirochaeta*	Curved spiral	5–250	*Spirochaeta plicatilis*	No	‐‐‐‐‐	Mollusk shell
*Treponema*	Tight spiral	1–20	*Treponema pallidum*	Syphilis	*Treponema pallidum*	Genital
				Periodontal disease	*Treponema denticola*	Oral cavity
				Yaws	*Treponema pertenue*	Mucous membrane
				Pinta	*Treponema carateum*	Mucous membrane
*Borrelia*	Sparsely wavy	10–35	*Borrelia anserina*	Relapsing fever	*Borrelia recurrentis*	Ixodes
				Lyme disease	*Borrelia burgdorferi*	Body louse
*Leptospira*	Hooked spiral	6–12	*Leptospira interrogans*	Leptospirosis	*Leptospira interrogans*	Rodents, mammals

Like with other pathogenic bacteria, a series of immune reactions occur when pathogenic spirochetes enter the body. The immune responses can be divided into innate and acquired immune responses. When pathogen‐associated molecular patterns (PAMPs) interact with pattern‐recognition receptors (PRRs), these PRRs include Toll‐like receptors (TLRs), nucleotide‐binding oligomerization domain (Nod), RIG‐like receptors (RLRs), C‐type lectin receptors (CLRs), and so on.^12^, ^13^ The innate immune system is activated, with macrophages, dendritic cells (DCs), and neutrophils helping to identify the pathogen and subsequently activating the complement system to eliminate nonspecific pathogens. In addition, specific pathogens are then killed by the acquired immune system. Although there is an obvious immune response after spirochete infection, the pathogenic bacteria cannot be completely cleared, which is why it leads to persistent infection *in vivo*. In this review, we will focus on the immune evasion mechanism of spirochetes in terms of antigen variation, complement inhibition, and immune interference, and summarize how pathogenic spirochetes escape from the strictly controlled immune system and colonize the host to affect humans and animals alike. A deeper understanding of the immune escape mechanisms of spirochetes will help us to better understand why it is so challenging to eradicate spirochete infections and ultimately provide new insights for developing effective vaccines against them in the future.

## 2 ANTIGENIC VARIATION

It is generally accepted that antigenic variation is a common pathogenic mechanism adopted by bacterial, protozoan, and fungal pathogens to cope with host identification and defense.^14^ It is commonly found in an evolutionary variety of obligate parasites, such as *Neisseria gonorrhoeae* (gonorrhea), *Giardia lamblia* (giardiasis or beaver fever), *Treponema pallidum* (syphilis), *Mycoplasma pulmonis* (mycoplasma pneumonia), *Borrelia recurrentis* (relapsing fever), or *Borrelia burgdorferi* (Lyme disease). In this part, we will consider *B*. *burgdorferi* as an example to illustrate the mechanism of antigen variation.

**FIGURE 1:**
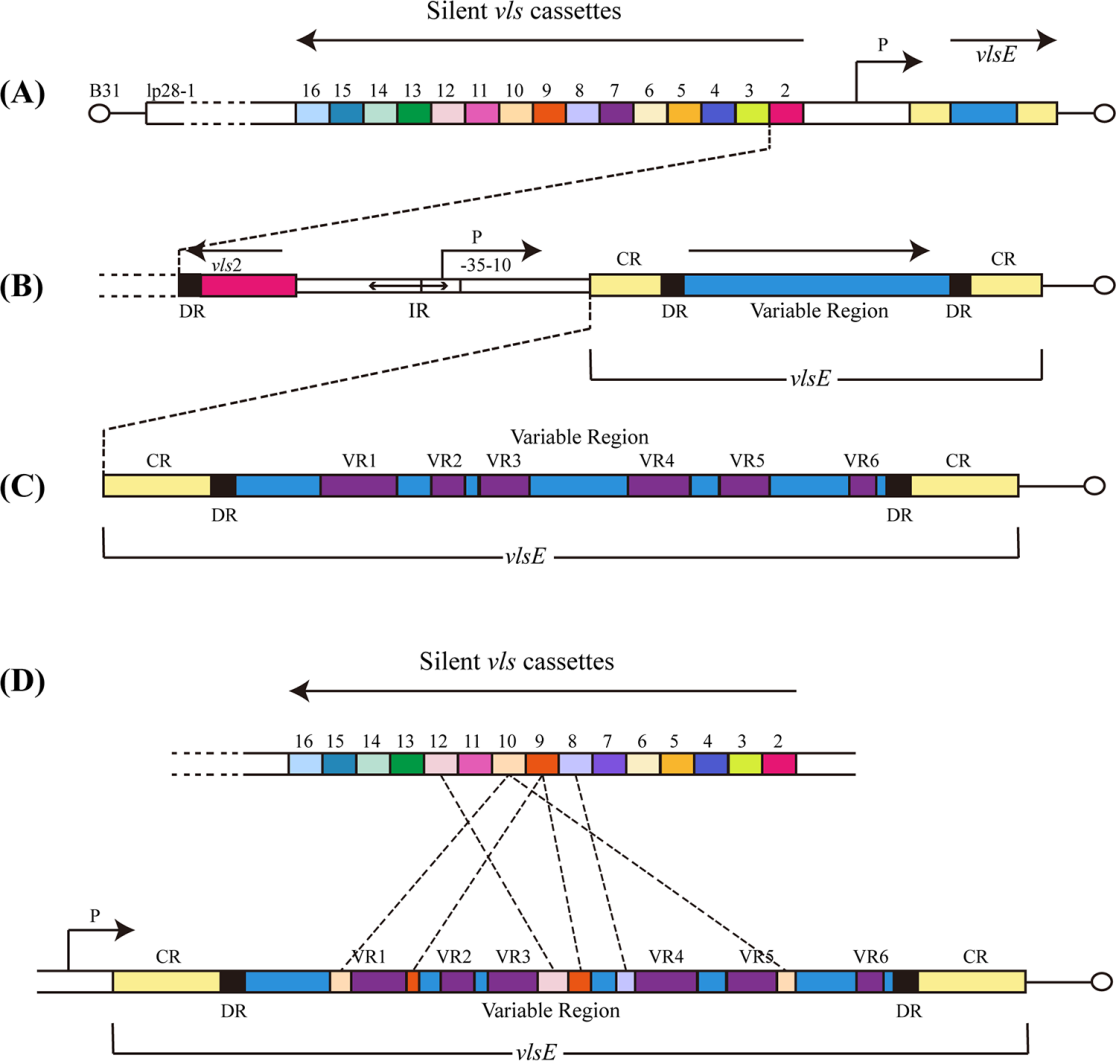
Schematic diagram of the *vls* system structure and recombination pattern. (A) In the *B*. *burgdorferi* of B31‐type strain, the *vls* locus is located on lp28‐1 and the *vlsE* gene is located near the telomere of the hairpin, 15 silent cassettes are located near and upstream of *vlsE*, but in the opposite direction (arrow). (B) The *vlsE* region is described in more detail. It is composed of the variable region (VR) and the constant region (CR), the variable region is flanked by 17‐bp direct repeats (DRs). To the left of *vlsE* is a part of the promoter (P), with the −10 and −35 inverted repeat sequences (about 100‐bp in length). (C) The central variable cassette region of *vlsE* is comprised of six variable regions (VR1–VR6) and six invariant regions (IRs). (D) In infected mammalian hosts, a random, unidirectional, and segmental combination of the *vlsE* protein‐coding site and the silent cassettes leads to differences in the expression of *vlsE* variants. Modified from Ref 16, 19, 30

Lyme disease is a multisystem infectious disease caused by *B. bugdorferi* and is the most common vector‐borne disease affecting humans in North America and temperate Eurasia. In the United States, Lyme disease is transmitted by hard‐bodied ticks of the genus *Ixodes*. Infected ticks will spread *B*. *burgdorferi* to humans during feeding, which can lead to a local infection (erythema migrans) at the site of the tick bite.^15^ The clinical manifestation is a chronic infection characterized by neuritis, arthritis, and myocarditis. *B. burgdorferi* has a highly evolved variable major protein (VMP)‐like sequence (*vls*) antigenic variation system, which possesses the ability to establish persistent infection by continuous variation of *vlsE*, a surface‐bound lipoprotein.^16^ The *vls* locus of B31 is located on the 28‐kb linear plasmid (lp28‐1). The *vls* locus comprises an expression site encoding the 35 kDa lipoprotein *vlsE* and a consecutive array of 15 silent *vls* cassettes (474–594 bp in length). The direction of these silent cassettes is opposite to the *vlsE* locus (Figure 1A).^17^ The *vlsE* locus and the silent *vls* cassettes are separated by a short intergenic region, and this intergenic space includes a 51‐bp inverted repeat (IR) sequence.^18^ Furthermore, a part of the promoter required for *vlsE* expression is located within this inverted repeat. The expression region of *vlsE* is composed of a central variable cassette, and the constant region (CR) is located on both sides of the central variable cassette. There are 17‐bp direct repeats (DR) at the junction of the variable and CRs and both ends of most silent cassettes (Figure 1B).^19^ The silent cassettes show high homology with the central variable cassette region of *vlsE* (90–96.1% nucleotide sequence identity). There are six variable regions and six invariant regions located in the central variable cassette region of *vlsE*, and most of the antigenic variation sequence differences are found in these six variable regions (Figure 1C).

Antigenic variation is a random combination of the *vlsE* protein‐coding site and the silent cassettes that lead to a difference in the expression of *vlsE* variants. In the *vls* system, *B*. *burgdorferi* can evade the host's acquired immunity via random, unidirectional, and segmental gene conversion.^16^, ^20^ Previous studies in infected mice^21^ or rabbits^22^ showed that while *vlsE* sequence variation occurs within 4 days and continues throughout infection, they could not be detected *in vitro* or the tick vector.^23^ This suggests that *vlsE* variants only exist in mammals. Although *vlsE* through segmental gene conversion provides a large number of diversity variable sequences during antigenic variation to evade immune surveillance, more about its variation mechanism is still unclear. The proposed model for *vlsE* antigenic conversion indicated^24^ that the *vlsE* central cassette regions are replaced by fragments of varied length and location in the silent cassettes. Bankhead^19^ performed an in‐depth analysis of the *vlsE* sequence changes and found that the *vls* antigenic variation system promotes varying length (short or long) recombination events in each cassette region. With the development of gene‐conversion events, other template‐independent changes also resulted in the amplification of *vlsE* variant sequences. The result of these cumulative changes is the generation of a new *vlsE* sequence with a mosaic structure (Figure 1D). Thus, these mutated antigens prevent recognition by the host immune system. Moreover, it has been well documented that the lack of *vlsE* or *vls* genes residing on lp28‐1 will cause the spirochete to lose its persistent infection. Other lp28‐1 non‐*vls* genes are not involved in the virulence, persistence, and recombination of *vlsE*. In other words, the *vls* locus and *vlsE* protein are key factors for immune escape and persistence of pathogens.^25^, ^26^, ^27^ Similarly, *T*. *pallidum* has a corresponding protein gene (TprK) that causes persistent infection through antigenic variation,^28^ but the mechanism is not yet known.

In recent years, some technical progress has been made in the study of the *vls* locus. These include the development of a new‐generation sequencing method^29^ for the analysis of *vlsE* recombination switches and the mini‐*vls* system^30^ for the genetic manipulation of the *vls* locus. Through this information, we have deepened our understanding of the antigenic variation mechanism of spirochetes. Although *vlsE* is flexible and variable in evolution, the locus has strictly conservative structural characteristics. These structures are indispensable in the process of antigenic variation. Therefore, whether we can inhibit the persistent infection by destroying the structure of the *vls* locus or using some genetic tools to achieve the effect of disease treatment remains to be further discussed. Understanding the definite mechanism behind the antigenic variation of spirochetes may lay the foundation for intervention measures to inhibit infection. For now, however, we still have a long way to go to overcome the obstacles of gene manipulation.

## 3 OVERCOMING THE ATTACK OF THE COMPLEMENT SYSTEM

**FIGURE 2:**
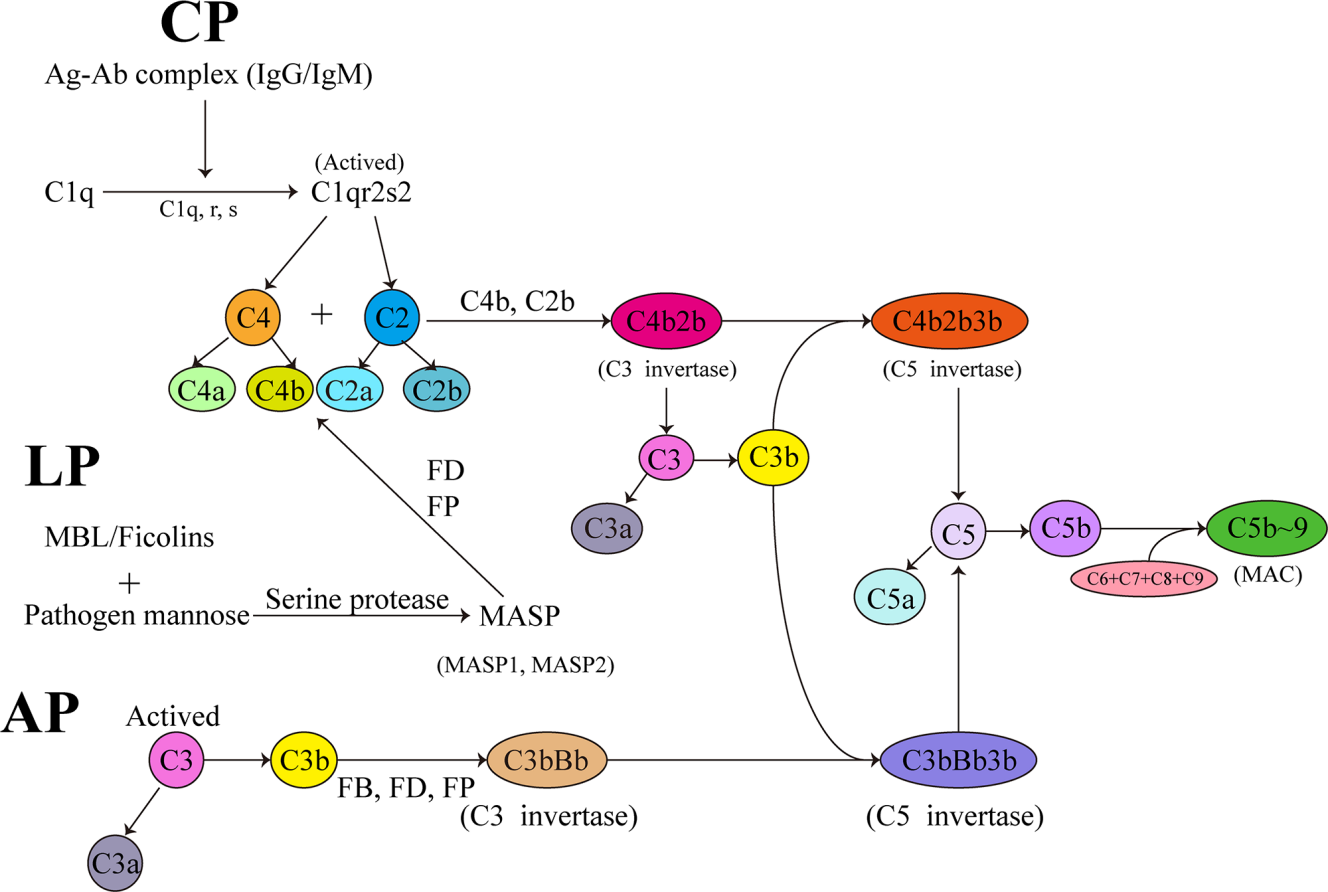
Schematic diagram of three activation pathways of the complement. There are three pathways to activate the complement system: classical pathway (CP), lectin pathway (LP), and alternative pathway (AP). All three pathways lead to the production of C3 invertase and C5 invertase, which cleave C3 and C5 respectively, and the C5 is then cleaved to form C5a and C5b, in that C5b combines with C6 and C7 to form a C5bC6C7 complex which is inserted into the cell membrane. The complex binds to C8 and joins with C9 to form a membrane attack complex (MAC). Finally, the cell starts to lyse. MBL, mannose‐binding lectin; MASP, MBL‐associated serine protease; FB, Factor B; FD, Factor D; FP, Factor D; MAC, membrane attack complex (C5b‐9)

The complement system is comprised of more than 50 glycoproteins, including plasma complement components, soluble proteins, membrane‐bound proteins, and complement receptors. In innate immunity, the complement system forms an important line of defense against microbial invasion. Actually, the complement system can be activated through three relatively independent and interrelated pathways to exert various biological effects such as regulating phagocytosis, cracking cells, mediating inflammation, regulating immune and clearing immune complexes. These three pathways are the classical pathway (CP), alternative pathway (AP), and lectin pathway (LP). All three pathways lead to the production of C3 invertase and C5 invertase that cleave C3 and C5, respectively, and pass through a common terminal pathway to form a membrane attack complex (MAC) (Figure 2). After activation of complement molecules, the complement components and surface substance of the pathogens form a complex, which ultimately promotes bacterial dissolution.^31^ In order to overcome the attack of the complement system, spirochetes take some evasion strategies that include acquiring host complement regulators, cracking complement components by binding of surface protein and Plg, and inhibiting complement activation by the interaction between the surface proteins of spirochetes and complement substances are introduced.

### 3.1 Recruitment of host complement regulators

The activation of the complement system requires complement factors to perform normal physiological functions. If complement regulation is out of control, a large number of the complement factors will be consumed, which will lead to a decrease in the body's resistance to infection and also cause severe inflammation or damage to its tissues and cells. Therefore, the role of complement regulators is particularly important. Common complement regulators are soluble regulators and membrane‐binding proteins. The former mainly includes C1 inhibitor (C1‐INH), C4b‐binding protein (C4BP), Factor I (FI), Factor H (FH), and factor H‐like‐1 (FHL‐1). The latter mainly includes complement receptor 1 (CR1), membrane cofactor protein (MCP), and decay‐accelerating factor (DAF). In addition, the regulators of the terminal activation sequence comprise vitronectin (Vn), clusterin (Cn), and some of the factor H‐related (FHR) proteins.^32^ Serum‐resistant strains of *Borrelia*,^33^, ^34^ *Leptospira*,^35^, ^36^and *Treponema* ^37^, ^38^ recruit the Factor H family, FHL‐1, FHR, and C4BP to inhibit the CP and AP. Different types of spirochetes have different surface‐binding proteins, which accelerate the inactivation of C3b and C4B by binding to these complement regulators.

Several *Borrelia* proteins such as CspA (CRASP‐1)^39^, ^40^ and CspZ (CRASP‐2)^41^, ^42^ can recruit FH/FHL‐1 to accelerate the inactivation of C3b. In addition, CspA binds the complement components C7 and C9 of the terminal pathway and blocks the assembly and membrane insertion of the terminal complement complex (TCC), thereby inhibiting the terminal complement pathway.^43^ ErpP (CRASP‐3), ErpC (CRASP‐4), and ErpA (CRASP‐5) have been discovered which combined with FHR‐1, FHR‐2, and partly with FHR‐5 to inhibit complement activation on the cell surface.^44^ Moreover, P43 (an outer membrane protein) can bind to C4bp and activate cofactor activity of factor I‐mediated cleavage of C4b.^34^ Among the *Leptospira* proteins, such as *L*. *interrogans* endostatin‐like outer membrane proteins A and B (LenA/LenB),^45^ *Leptospira* immunoglobulin‐like proteins A and B (LigA and LigB),^35^ Lsa33 (LIC11834), and Lsa25 (LIC12253)^46^ can also bind to FH, FHL‐1, FHR‐1, and C4BP at different sites *in vitro* and regulate the complement activation pathway. Furthermore, *Leptospira* complement regulator‐acquiring protein A (LcpA) is capable of binding vitronectin via regulating the terminal pathway to reduce MAC deposition.^47^ Different from *Borrelia* and *Leptospira*, Factor H binding protein B (FhbB) of *Treponema* can reduce complement factor activity by interacting with FH.^48^ Taken together, all of the above examples illustrate the mechanism of immune evasion mediated by spirochetes through the recruitment of complement regulators.

### 3.2 Cleavage of complement components

When pathogenic microorganisms invade the body, the inactivated complement components in human serum will be activated in the complement system by antigen–antibody complexes. The activated complement components possess enzymatic activity, which can mediate the immune response and inflammatory response, lyse C3b to combine with bacteria to promote phagocytosis of phagocytes, or form membrane attack complex (MAC) to lyse target cells and cause cell death. Spirochete surface proteins, as a kind of important factor to resist innate defense, can cleave the complement factors by binding to plasmin (Pla).

Based on published literature, we know that several plasminogen/plasmin (Plg/Pla) receptors of *Borrelia* can degrade the complement components C3, C3b, C4b, and C5 via binding to surface proteins such as the Erp family,^49^ BBA70 protein,^50^ CspA,^39^ and CspZ^51^ proteins. Furthermore, the HcpA protein, as a surface protein of *B. recurrentis* can also reduce the deposition of C3b via Pla binding.^52^ Similar to *Borrelia*, the surface Plg/Pla of *Leptospira* results in downregulation of C3b, C4, and C5 by capturing LigA and LigB,^53^ Lsa23 (LIC11360),^54^ Lsa30,^55^ and elongation factor thermo unstable (EF‐Tu),^56^ among other proteins. However, thus far, it is still a challenge to determine the biological correlation between the surface protein of spirochetes and Plg/Pla‐binding protein.

### 3.3 Inhibition of complement activation

The complement system can be activated by three pathways. Each of the complement components from these three pathways is critical, and the absence or downregulation of even one component will inhibit complement activation. Expect the above two points about the recruitment of complement regulators and the cleavage of complement components, the direct effect of spirochete surface protein on complement factors can also inhibit activation of the complement system.

There is a new mechanism for downregulating the CP of complement. The surface protein BBK32 expressed by *Borrelia* can block C1r to inhibit the C1 complex composed of C1q, C1r, and C1s. BBK32 acts directly on C1r and inhibits the autocatalysis of C1r in the C1 complex, which fails activation of the CP. However, the LP and AP are not affected, because they lack the targeting factor for BBK32.^57^ Therefore, BBK32 can be used as an effective inhibitor of the CP by interfering with C1r. At the same time, BGA66, BGA71, and CspA inhibit complement activation by interfering with the terminal pathway. They interact directly with C7, C8, and C9, and may affect MAC assembly by preventing c5b‐8 complexes from inserting into the target cell membrane correctly and also inhibit the ability of C9 to aggregate with subsequent factors.^43^, ^58^ Besides, early work by Caine et al.^59^ established that the outer surface protein C (OspC) from *B*. *burgdorferi* has complement resistance, which can inhibit CP and LP by competing with complement protein C2 for C4B binding and survive in the bloodstream.

Recently, two new hypothesized proteins of *Leptospira* encoded by the LIC12587^60^ and LIC13259^61^ genes have been reported. Recombinant protein LIC12587 interacts with C7, C8, and C9 components of the complement system in a dose‐dependent manner, reducing the sterilization effect of the complement. Binding to C9 may result in inhibition of C9 polymerization and interfere with the formation of MAC. Similarly, recombinant protein LIC13259 can recruit and interact with vitronectin, C7, C8, and C9 from normal human serum. Cavenague et al. showed that the binding of rLIC13259 with C8 and vitronectin was inhibited gradually with the increase of heparin concentration, indicating that the interaction with vitronectin occurred through the heparin domain. Most interestingly, the interaction between rLIC13259 and C9 can prevent C9 polymerization from inhibiting MAC formation. To summarize, by degrading or downregulating the complement components, the pathogenic spirochetes can limit activation of the complement system, and finally achieve the goal of immune escape.

## 4 INTERFERENCE WITH IMMUNE REGULATION

The immune response is that immune cells recognize, activate, proliferate, and produce immune substances under the stimulation of antigens, so as to mediate specific immune effects and finally eliminate invading pathogens. The body can maintain a relatively stable state through appropriate immune regulation. As is well known, immune responses can be divided into innate immunity and adaptive immunity. First, how does the immune system detect the existence of pathogens? There are multiple receptors on the surface of mammalian cells, which can recognize specific molecular characteristics of pathogens. These characteristics are called pathogen‐associated molecular patterns (PAMPs),^13^ including bacterial lipopolysaccharides (LPS), lipoproteins, peptidoglycan, and flagellar proteins. The host receptors are called pattern recognition receptors (PRRs) that interact with PAMPs to initiate a series of intracellular signal cascades that trigger an innate immune response and subsequent adaptive immune responses. This process initiates the production of a series of cytokines, including polymorphonuclear neutrophils, monocytes, macrophages, dendritic cells, and natural Killer T Cells, which lead to inflammation in the body. Toll‐like receptors (TLRs) are the main recognition receptors of spirochetes. TLRs can recognize spirochetal membrane components, which play a major role in the inflammatory induction of spirochete infections. Interestingly, spirochetes (*Treponema*, *Borrelia*) as gram‐negative bacteria, despite the lack of surface LPS, can still be attacked by TLRs recognition and the immune system.^62^ Next, we will focus on the interference of innate immune cells and their surface lipoproteins on immune regulation.

### 4.1 Impairment of bactericidal function of polymorphonuclear neutrophils (PMNs)

Human PMNs are one of the fastest and earliest immune cells to react during acute infection and possess a variety of microbicide mechanisms. After TLRs recognize and activate neutrophils, hydrolases and strong oxidizing bactericidal substances, such as H2O2, myeloperoxidase, and antimicrobial peptides will be released, which will eventually lead to the elimination of bacteria through the phagocytosis, hydrolysis, and oxidative burst of neutrophils. Furthermore, a study^63^ has proposed a novel pathogen‐killing mechanism of neutrophils. When bacterial or fungal species are activated, the nuclear DNA of neutrophils will be released outside of the cell. These DNA structures are called neutrophils extracellular traps (NETs) that can kill pathogens without relying on phagocyte uptake and degranulation.

These mechanisms may be important for early infection and transmission of spirochetes. Surprisingly, *Leptospira* is hardly phagocytized in neutrophils and can be killed only in the presence of specific antibodies. Vieira et al. proved that Leptospiral outer membrane protein LipL21 can be used as a myeloperoxidase inhibitor to inhibit the oxidation and chlorination activity of myeloperoxidase without interfering with neutrophil degranulation, which is conducive to the survival of *Leptospira* in the host.^64^ Likewise, in the *B*. *burgdorferi* lipoprotein BBA57 experiment, infected mice with wild‐type or BBA57 mutant were genetically analyzed that BBA57 mutant mice reduced the expression levels of various pro‐inflammatory factors such as IL‐1β, IL‐6, TNF, or the chemokines Ccl3. On the other hand, the transcription level mediated by PRRS and downstream signaling molecules is also downregulated including Tlr2, Myd88, and Nlrp3. The results suggest that BBA57 can inhibit the activation of innate immune cells.^65^ In addition, spirochetes seem to release significant nucleases to degrade the nuclear DNA of neutrophils and prevent them from being trapped and killed by NETs, which helps to spread in the host.^66^ However, these specific mechanisms remain to be further studied.

### 4.2 Anti‐phagocytosis and pro‐apoptosis effect on monocytes and macrophages (M/M)

Other than neutrophils, M/M also provides innate immune protection to the host, especially in the early stages of infection. Spirochete lipoproteins bind to CD14 on the M/M membrane and activate the nuclear transcription factor‐κB (NF‐κB) pathway.^67^, ^68^ Spirochetes stimulate M/M to produce cytokines such as IL‐1, IL‐10, IL‐6, and TNF‐α, which is one of the main immune responses under TLRs mediation.^69^, ^70^, ^71^ Thus, we speculate that spirochetes may evade the immune response by resisting phagocytosis or promoting apoptosis of immune cells.

In recent years, the mechanism by which leptospirosis induces apoptosis of macrophages has generated much discussion. Two likely mechanisms are discussed below to explain the apoptotic process. Hu et al.^72^ demonstrated that *Leptospira* induced apoptosis through mitochondrial damage in macrophages. The release of apoptosis‐inducing factor (AIF) and endonuclease G (EndoG) from the mitochondria and subsequent nuclear translocation can lead to nuclear DNA breakage and apoptosis. During infection, caspase‐8 and Bid protein were activated, and highly reactive oxygen species (ROS) led to Akt (or protein kinase B, PKB) dephosphorylation. More specifically, Bid‐mediated mitochondrial release of AIF and/or EndoG and nuclear translocation are both major mechanisms of *Leptospira*‐induced apoptosis in macrophages, and this process is regulated by both caspase‐8 and ROS‐Akt signal pathways. Another new study reported that LPS on the surface of *Leptospira* promotes the expression of Fas and FasL in macrophages and cell membrane migration. The newly discovered recombinant protein *L. interrogans* LB047 gene is the only protein captured by mouse and human Fas proteins, which is significantly upregulated in macrophage infection. With the participation of transcription factors c‐Jun and ATF2, c‐Jun N‐terminal kinase (JNK) and p38 mitogen‐activated protein kinase (MAPK) signal pathways are activated by TLR2, and finally, macrophage apoptosis is induced by Fas/FasL‐caspase‐8/‐3 pathway.^73^ On the other hand, by recognizing CD14 and/or TLR2 on the cell surface, the outer membrane protein Tp92 of *T. pallidum* induces apoptosis of THP‐1 cells via the pro‐caspase‐1 pathway. At the same time, the protein induces apoptosis of THP‐1 cells by the protein kinase 1/caspase‐8/caspase‐3 pathway under the receptor interaction.^74^

Results from these related studies have helped us to define the anti‐phagocytosis and pro‐apoptotic mechanism of spirochetes and provide a new perspective.

### 4.3 Inhibition of dendritic cells (DCs) and natural killer T (NKT) cells

Dendritic cells and NKT cells are also essential cells in the innate immune system and play an irreplaceable role in eliminating bacterial infection. DCs are the most powerful antigen‐presenting cells, which can induce naive T cells and act as the sentinel of immune responses. NKT cells can nonspecifically modulate Th1/Th2. They provide a bridge between innate and adaptive immunity.

Inhibiting the function of these cells may be one of the mechanisms of spirochete immune escape. TLR‐mediated pro‐inflammatory effects can produce a large number of cytokines,^75^ the most special of which is IL‐10. Because it is different from other cytokines such as IL‐1 and IL‐12, IL‐10^76^ is thought to have the ability to downregulate the inflammatory response via the TLR pathway. It can help to fight against spirochetal cell wall infection and any possible chronic effects such as arthritis.^77^ In a mouse experiment, Zhang et al.^78^ found that when *Leptospira* and TLR2 agonist Pam3csk4 were injected into hamsters, the IL‐10 produced in the tissues of mice injected with *Leptospira* and Pam3csk4 was increased as compared with the group injected with *Leptospira* alone. Similarly, the IL‐10 level of TLR2‐deficient mice was lower than that of wild‐type mice. This suggests the use of TLR2 agonists to induce IL‐10 production, IL‐10 can downregulate the inflammatory system, thereby weakening the inflammatory response of the body. LipL32, as a major outer membrane protein of pathogenic *Leptospira*, is a TLR2 agonist and can induce a strong antibody response.^79^ Like *Leptospira*, *B*. *burgdorferi* can also induce IL‐10 to inhibit the production of inflammatory mediators by M/M and/or DCs in mice, and reduce the inflammatory response.^76^, ^80^

Regarding NKT cells, an important example is that spirochetes can directly interfere with NKT cells, which respond to CD1d glycolipids on the surface of spirochetes such as *B*. *burgdorferi*.^81^ Although the exact mechanism of interference is still unclear, further studies are needed to understand the possible interactions between spirochetes and NKT cells.

## 5 PERSISTENT INFECTION

The fibrinolytic (or Plg/Pla) system is an enzyme cascade consisting of many proteases and inhibitors that are involved in the production and regulation of Pla. Plg is transformed into Pla by tissue‐type Plg activator (tPA) or urokinase‐type Plg activator (uPA) in the fibrinolytic system. Pla, a broad‐spectrum serine protease is the core component of the fibrinolysis system, and its main function is the degradation of fibrinolytic proteins. Several studies have proved that spirochetes such as *Borrelia*, *Leptospira*, and *T. denticola* can bind Plg on their outer surface. Plg appears to combine with protein receptors through its kringle domains. As a Plg/Pla binding site, the lysine residue of spirochete receptors can induce the expression and/or release of Plg activators (tPA or uPA), thereby favoring conversion of surface‐bound Plg into Pla.^82^ These spirochetes with Pla activity will cause proteolysis of fibronectin and laminin, which are important parts of ECM and basal membranes. The result is to promote bacterial invasion of cells and transmission. In addition to binding Plg, spirochetes can also stimulate human monocytes to secrete Pla activators that will be helpful to form the Pla on the bacterial surface.^83^ On the other hand, pathogens can also indirectly promote the fibrinolytic system by stimulating endothelial cells to secrete matrix metalloproteinases (MMPs).^84^

There are different types of outer membrane proteins in spirochetes that can combine with Plg to produce Pla. For instance, *Borrelia* species have the outer surface proteins A^85^ and C^86^ (OspA and OspC). These two proteins attach to the intestinal tract of ticks and infect mammalian hosts to colonize and survive. Likewise, in relapsing fever disease, CbiA showed the ability to promote complement binding and inhibit the activation of regulators through interaction with Pla, which would prevent the complement system from attacking pathogens.^87^ Some *Leptospira* Plg receptors such as the major outer membrane protein LenA,^88^ LipL46,^89^ OmpL1,^90^ and OmpA (Lsa66),^91^ or *T*. *denticola* chymotrypsin‐like protease were also discovered that invades host cells by degrading the ECM and basement membrane components.^92^

From the above conclusion, we found that interaction between spirochetes and the host fibrinolytic system provides the bacterial membrane‐related proteins with hydrolytic activity. This property contributes to the degradation of ECM or basal membrane components and epithelial or endothelial tissue penetration, thereby promoting the bacterial invasion of the host, immune escape, and transmission. Recently, a new study reported that *Leptospira* has two newly developed recombinant proteins, the gene loci of which are LIC11711^60^ and LIC13259.^61^ Both these recombinant proteins are capable of acquiring Plg from normal human serum and translating them into enzyme‐active Pla under the effect of the Plg activator. The discovery of these new Plg‐binding proteins will play an important role in the invasion and colonization of hosts. The introduction of more new recombinant proteins may deepen our understanding of the fibrinolytic system and the immunopathogenesis of spirochetes.

## 6 CONCLUSION

Spirochetes are prokaryotic microorganisms between bacteria and protozoa. It not only has the similar structure and biological characteristics of bacteria, including cell wall, binary fission, amorphous nucleus, and sensitivity to antibiotics (penicillin), but also are soft as protozoa. It can move flexibly by bending and contracting the elastic filaments between the cell wall and the cell membrane, but it differs from other pathogens such as fungi, viruses, and parasites in that it does not have a complete cell structure, nor is it a strictly intra‐host parasitic organism. However, these pathogens all have a similar set of self‐protection and independent immune evasion mechanisms that interact with the immune capacity of the host organism to form a certain balance, which is the result of their long‐term co‐evolution. Under the surveillance of the powerful and hostile immune system, spirochetes have developed many strategies to resist the detrimental effects of immune factors. In this work, we summarized several escape mechanisms of spirochetes, including antigenic variation, complement inhibition, and immune interference to subvert the immune response. All the above‐mentioned measures increase the possibility of spirochete survival in the host and lead to a persistent chronic infection. As we know, the *vls* locus has strictly conservative structural characteristics. With the successful construction of a mini‐*vls* system, we speculate whether we can block the recombination switch or destroy the structure of the *vls* site or use other genetic tools to inhibit persistent infection, to achieve the effect of successful disease treatment that remains to be further investigated. There are still several challenges concerning gene manipulation as a potential therapy. Moreover, we found that many escape mechanisms of spirochetes are closely associated with the spirilla surface proteins. In recent years, scientists have also developed some candidate vaccines for spirochetes using surface proteins. Our future studies are aimed at identifying more immune‐related proteins to contribute to the development of vaccines against spirochetes.

## CONFLICT OF INTEREST

The authors declare that the research was conducted in the absence of any commercial or financial relationships that could be construed as a potential conflict of interest.

## AUTHOR CONTRIBUTIONS

Jielite Huang drafted the manuscript. Jinlin Chen and Yafeng Xie modified the manuscript. Zhuoran Liu conceived the idea.

## Data Availability

Data sharing is not applicable to this article as no new data were created or analyzed in this study.
